# TLR4 sensing of IsdB of *Staphylococcus aureus* induces a proinflammatory cytokine response via the NLRP3-caspase-1 inflammasome cascade

**DOI:** 10.1128/mbio.00225-23

**Published:** 2023-12-19

**Authors:** Juan José Izquierdo Gonzalez, Md Faruq Hossain, Jolanda Neef, Erin E. Zwack, Chih-Ming Tsai, Dina Raafat, Kevin Fechtner, Luise Herzog, Thomas P. Kohler, Rabea Schlüter, Alexander Reder, Silva Holtfreter, George Y. Liu, Sven Hammerschmidt, Uwe Völker, Victor J. Torres, Jan Maarten van Dijl, Christopher H. Lillig, Barbara M. Bröker, Murty N. Darisipudi

**Affiliations:** 1 Institute of Immunology, University Medicine Greifswald, Greifswald, Germany; 2 Institute for Medical Biochemistry and Molecular Biology, University Medicine Greifswald, Greifswald, Germany; 3 Department of Medical Microbiology, University of Groningen, University Medical Center, Groningen, the Netherlands; 4 Department of Microbiology, New York University Grossman School of Medicine, New York, USA; 5 Department of Pediatrics, Division of Infectious Diseases, University of California San Diego, La Jolla, California, USA; 6 Department of Microbiology and Immunology, Faculty of Pharmacy, Alexandria University, Alexandria, Egypt; 7 Department of Molecular Genetics and Infection Biology, Interfaculty Institute for Genetics and Functional Genomics, Center for Functional Genomics of Microbes, University of Greifswald, Greifswald, Germany; 8 Imaging Center of the Department of Biology, University of Greifswald, Greifswald, Germany; 9 Department of Functional Genomics, Interfaculty Institute for Genetics and Functional Genomics, Center for Functional Genomics of Microbes, University of Greifswald, Greifswald, Germany; University of Würzburg, Würzburg, Germany; Philipps-Universität Marburg, Marburg, Germany

**Keywords:** cytokines, innate immunity, IsdB, *Staphylococcus aureus*, TLR4, NLRP3 inflammasome

## Abstract

The iron-regulated surface determinant protein B (IsdB) of *Staphylococcus aureus* is involved in the acquisition of iron from hemoglobin. Moreover, IsdB elicits an adaptive immune response in mice and humans. Here, we show that IsdB also has impact on innate immunity. IsdB induces the release of proinflammatory cytokines, including IL-6 and IL-1β, in innate immune cells of humans and mice. *In silico* analysis and thermophoresis show that IsdB directly binds to TLR4 with high affinity. TLR4 sensing was essential for the IsdB-mediated production of IL-6, IL-1β, and other cytokines as it was abolished by blocking of TLR4-MyD88-IRAK1/4-NF-κB signaling. The release of IL-1β additionally required activation of the NLRP3 inflammasome. In human monocytes infected with live *S. aureus*, IsdB was necessary for maximal IL-1β release. Our studies identify *S. aureus* IsdB as a novel pathogen-associated molecular pattern that triggers innate immune defense mechanisms.

**IMPORTANCE:**

The prevalence of multidrug-resistant *Staphylococcus aureus* is of global concern, and vaccines are urgently needed. The iron-regulated surface determinant protein B (IsdB) of *S. aureus* was investigated as a vaccine candidate because of its essential role in bacterial iron acquisition but failed in clinical trials despite strong immunogenicity. Here, we reveal an unexpected second function for IsdB in pathogen-host interaction: the bacterial fitness factor IsdB triggers a strong inflammatory response in innate immune cells via Toll-like receptor 4 and the inflammasome, thus acting as a novel pathogen-associated molecular pattern of *S. aureus*. Our discovery contributes to a better understanding of how *S. aureus* modulates the immune response, which is necessary for vaccine development against the sophisticated pathogen.

## INTRODUCTION

*Staphylococcus aureus* (*S. aureus*) is an opportunistic Gram-positive pathogen that can cause life-threatening diseases and is regarded as one of the major threats to global health (1, 2). The treatment of staphylococcal infections is hampered by a continuously high prevalence of methicillin-resistant *S. aureus*. Therefore, new strategies for the control of *S. aureus* are urgently needed, including novel therapeutic options (1–4) and effective vaccines (5, 6).

Innate immune cells recognize conserved microbial structures by virtue of pattern recognition receptors (PRRs) such as Toll-like receptors (TLRs) and NOD-like receptors (NLRs) (7, 8). *S. aureus* possesses a wide variety of virulence factors—toxins, enzymes, and cell wall components—that contribute to its pathogenesis and evasion from the host’s immune response (8). More recently, it was reported that PRRs can sense such species-specific virulence factors of *S. aureus*. For instance, the pore-forming toxins leukocidin F (9) and phenol-soluble modulins (PSMs) directly activate TLR4 (10). TLR activation commonly leads to the nuclear factor (NF)-κB-dependent production of proinflammatory cytokines such as IL-6, which promote pathogen clearance (11, 12). In addition, *S. aureus* is known to activate the NLR-containing protein 3 (NLRP3) inflammasome and induce caspase-1-dependent interleukin (IL)-1β release (13, 14). This is exemplified by pore-forming toxins, such as α-hemolysin (Hla) and LukAB, which modulate the host immune response by activating NLRP3 (15, 16).

Iron is vital for *S. aureus* survival in the host, but in homeostasis, there is virtually no free iron available (17, 18). In consequence, the microorganism employs toxins such as hemolysins and leukocidins (19, 20) to lyse erythrocytes and scavenges the iron from the released hemoglobin (Hb) through the iron-regulated surface determinant (Isd) protein system (21–23). Of all the proteins of the Isd system, IsdB is most strongly upregulated by *S. aureus* under iron-limited conditions (24). This cell wall-anchored protein is the main bacterial receptor of Hb, which binds with high affinity (25–27). IsdB possesses two specific iron uptake domains called near-iron transporter (NEAT) 1 and 2. NEAT1 allows high-affinity binding to Hb, while the NEAT2 domain acquires the heme group from Hb (26–28). *S. aureus* mutants lacking IsdB are strongly attenuated when free iron is limited (25, 29, 30). IsdB also mediates adhesion of *S. aureus* to host cells via integrin GPIIb/IIIa and through extracellular matrix component vitronectin (31–35). Since IsdB is vital for *S. aureus* and individuals that are colonized by *S. aureus* mount a significant IgG response against the bacterial protein, IsdB has been explored as a vaccine candidate (36, 37). The vaccinated individuals developed high antibody titers against IsdB that was administered without adjuvant, confirming that IsdB induces an adaptive immune response (37). However, it remained largely unknown if IsdB also influences the innate immune response. To explore a possible influence of IsdB on innate immunity, we first studied the direct effect of IsdB on innate immune cells *in vitro* and discovered that IsdB induces the release of inflammatory cytokines, especially IL-6 and IL-1β. We hypothesized that IsdB triggers these host innate immune defense mechanisms by engaging PRRs and activating the inflammasome. Analysis of signaling pathways revealed that IsdB binds and activates TLR4 and induces the NLRP3 inflammasome. In innate immune cells infected with live *S. aureus*, IsdB was necessary for maximal IL-1β production. We have thus uncovered an additional role for IsdB beyond iron acquisition from Hb and attachment to host cells. These findings represent a new facet in the pathogen-host relationship of *S. aureus* that should be considered in future vaccine development.

## RESULTS

### IsdB induces the production of cytokines *in vitro*

**Fig 1:**
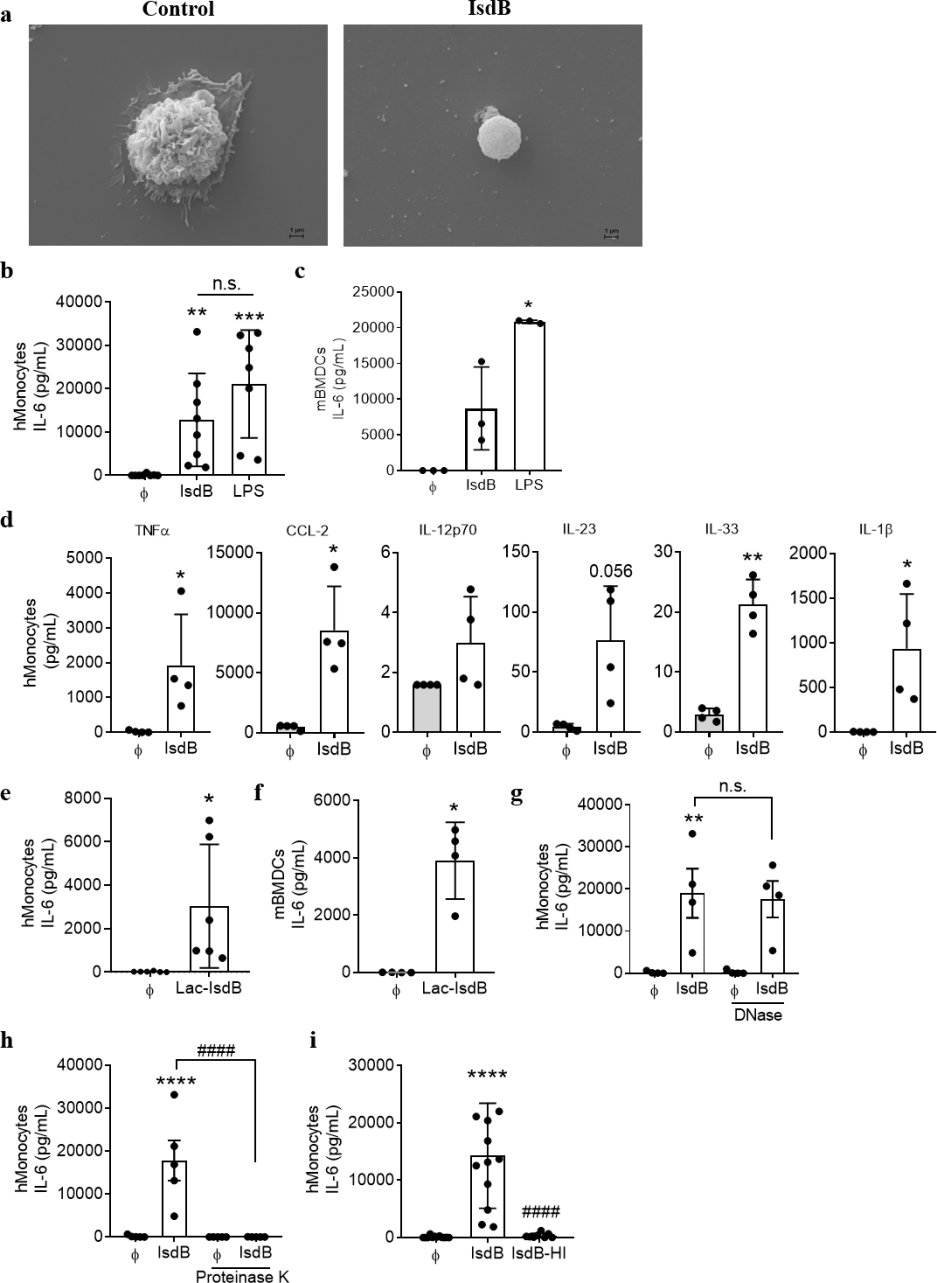
IsdB induces proinflammatory cytokine release in human monocytes and murine bone marrow-derived dendritic cells. (**a**) Scanning electron micrographs of primary human monocytes treated with or without IsdB (10 µg/mL) for 24 h. Representative images of monocytes from one donor are shown. Activated monocytes show membrane blebs and disintegrated membranes as compared with untreated cells. Scale bars = 1 µm. (**b**) Human monocytes or (**c**) mBMDCs were left unstimulated or stimulated with IsdB (10 µg/mL) or LPS (100 ng/mL) for 24 h (**b**) (*n* = 8) or 6 h or LPS (1 ng/mL) (**c**) (*n* = 3). Levels of IL-6 in the supernatants were quantified by Enzyme-linked Immunosorbent Assay (ELISA). (**d**) Supernatants from b were analyzed for indicated cytokines by a bead-based multiplex assay (*n* = 4). (**e**) Human monocytes or (**f**) mBMDCs were stimulated with Lac-IsdB (10 µg/mL) for 24 h (*n* = 6) or 6 h (*n* = 4), respectively. Levels of IL-6 in the supernatants were quantified by ELISA. (**g–i**) Primary human monocytes were stimulated with either IsdB (10 µg/mL), DNase-pre-treated IsdB, *n* = 4 (**g**), proteinase K-pre-treated IsdB, *n* = 5 (**h**), or heat-inactivated IsdB (IsdB-HI), *n* = 11 (**i**), for 24 h, and IL-6 release was measured in the supernatants by ELISA. Data are depicted as mean ± SEM of indicated biological replicates (“*n*”) performed in technical duplicate or triplicate. Each point represents one donor (human monocytes) or biological replicate (mBMDCs). One-way Analysis of Variance (ANOVA) was utilized to determine statistical significance in b, c, and i. Two-way ANOVA was utilized to determine statistical significance between the groups in g and h. The paired *t*-test was utilized to determine statistical significance in d, e, and f. *P < 0.05, **P < 0.01, ***P < 0.001, and ****P < 0.0001 represent IsdB or LPS vs untreated cells. *^####^P* < 0.0001 represents IsdB vs IsdB + PK or IsdB-HI. DNase, deoxyribonuclease; HI, heat inactivated; n.s., non-significant; PK, proteinase K. ϕ represents respective controls or unstimulated cells.

To study the effect of IsdB on innate immune cells, we initially incubated human monocytes with recombinant IsdB (hereafter referred to as IsdB) for 24 h. Scanning electron microscopy (SEM) of untreated cells showed large ruffle-like structures on the surface, which is a characteristic feature of resting monocytes. In contrast, IsdB-treated cells displayed disorganized membranes with blebs, which is a sign of activation (Fig. 1a). Next, we examined whether this monocyte activation translates into cytokine release. Indeed, IsdB induced the secretion of IL-6 in a dose- and time-dependent manner with robust IL-6 production at an IsdB concentration of 10 µg/mL (Fig S1a and S1b). IL-6 release was observed in the monocytes of all tested blood donors (Fig. 1b) as well as in murine bone marrow-derived dendritic cells (mBMDCs) (Fig. 1c). In both cell types, IsdB induced the release of approximately half as much IL-6 as plateau concentrations of lipopolysaccharide (LPS), a prominent inducer of inflammation. Next, we tested a broader cytokine panel and found that IsdB induced the release of several proinflammatory cytokines, including TNFα, CCL2, IL-23, IL-33, and IL-1β (Fig. 1d). The concentration of IL-12 was very low and did not significantly increase in response to IsdB treatment.

Since we had produced the recombinant IsdB in *Escherichia coli*, we took care to exclude the effects of possible LPS contamination. We rigorously depleted LPS from the purified preparations of recombinant IsdB by matrix-based affinity chromatography. When we added IsdB to immune cells at a concentration of 10 µg/mL, the maximal final concentration of LPS was below 15 pg/mL. At this concentration, LPS did not induce IL-6 on its own and it did not add significantly to the IsdB-induced cytokine release (Fig. S2). We also produced IsdB in an LPS-free system, *Lactococcus lactis* (hereafter referred to as Lac-IsdB). Lac-IsdB also triggered IL-6 release in both monocytes and mBMDCs (Fig. 1e and f). To exclude an impact of microbial genomic DNA contamination, which could act as a PAMP, we pre-treated the IsdB preparation with deoxyribonuclease (DNase) for 30 minutes (min) at 37°C but observed no effect on IL-6 release (Fig. 1g). In contrast, proteinase K as well as heat treatment of IsdB (95°C for 30 min) abrogated the release of IL-6 (Fig. 1h and i) and all other cytokines (Fig. S3).

### IsdB-induced cytokine release depends on the TLR4-MyD88 axis

**Fig 2:**
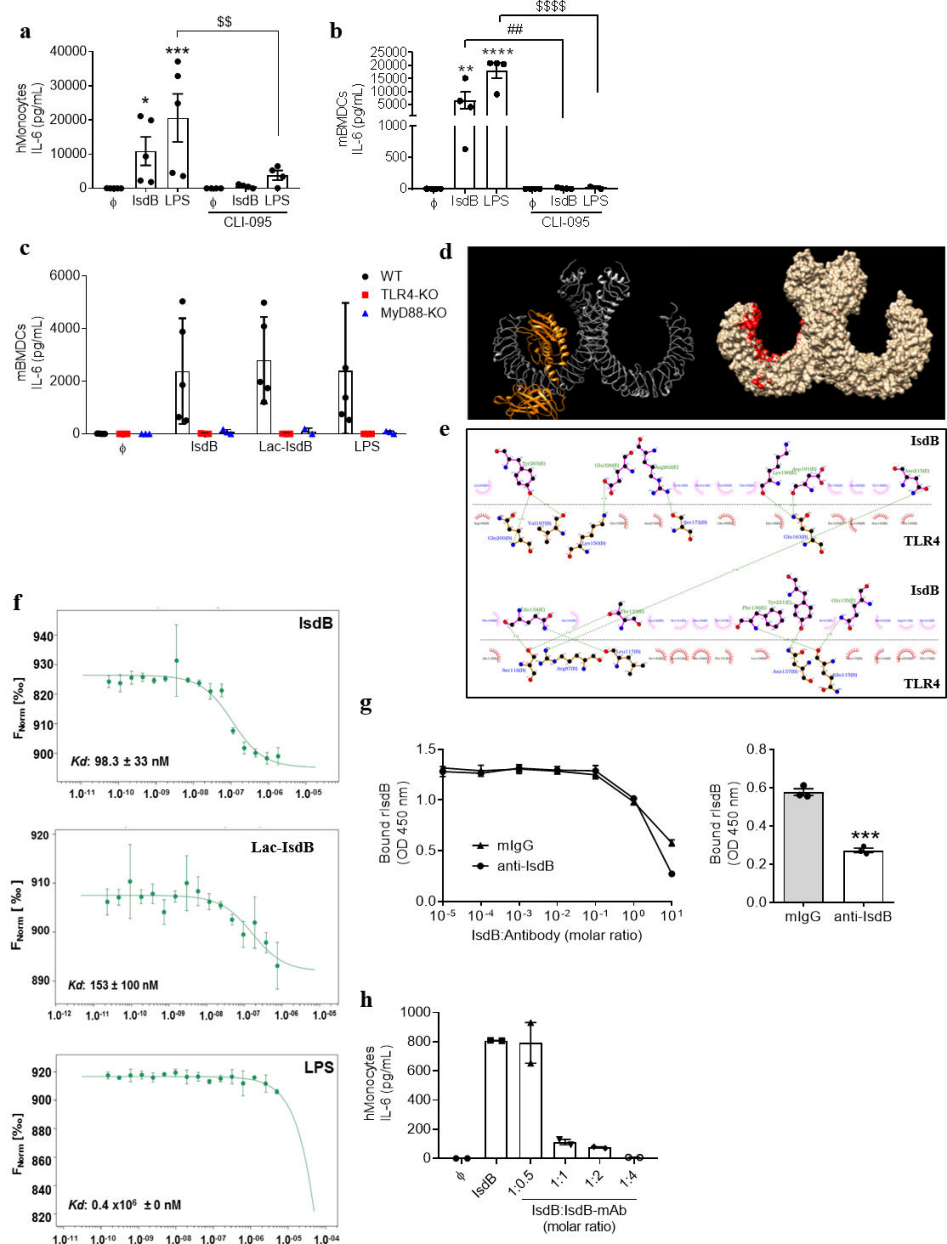
IsdB induces proinflammatory cytokine release via the TLR4-MyD88 signaling cascade. (**a**) Human monocytes were left untreated or pre-treated with CLI-095 (1 µM) for 45 min before treatment with either IsdB (10 µg/mL) or LPS (100 ng/mL) for an additional 24 h. *n* = 5. Cell-free supernatants were analyzed for human IL-6 by ELISA. (**b**) Wild-type (WT) mBMDCs were left untreated or pre-treated with CLI-095 (1 µM, 45 min) prior to treatment with IsdB (10 µg/mL) or LPS (1 ng/mL) for an additional 6 h. *n* = 4 except for the LPS + inhibitor group (*n* = 2). (**c**) WT, TLR4-, and MyD88-KO mBMDCs were left untreated or stimulated with IsdB (10 µg/mL), Lac-IsdB (10 µg/mL), or LPS (1 ng/mL) for 6 h. Cell-free supernatants were analyzed for the release of mouse IL-6 by ELISA. *n* = 5 WT and *n* = 3 TLR4-KO and MyD88-KO. (**d**) Molecular docking of hTLR4 and IsdB: red surface on hTLR4 represents the areas of interaction with IsdB. A representative hTLR4-IsdB complex (molecular model 2) demonstrates potential recognition. (**e**) Dim plot analysis represents non-covalent hydrophobic interactions between amino acid residues in the TLR4/IsdB complex. Dotted lines show hydrogen bonds. (**f**) Microscale thermophoresis (MST) analysis between recombinant human TLR4 (rhTLR4) and IsdB or Lac-IsdB or LPS: IsdB and Lac-IsdB shows strong binding toward rhTLR4 with an estimated dissociation constants (Kds) of 98.3 and 153 nM, respectively. In contrast, LPS failed to exhibit high-affinity binding in this assay due to the absence of MD-2. Kd was calculated using NanoTemper Affinity Analysis software. *n* = 3. (**g**) An ELISA plate was coated overnight at 4°C with recombinant TLR4 (0.5 µg/mL) and incubated with either IsdB (1 µg/mL) or IsdB preincubated with increasing molar ratios of the indicated antibodies, at Room Temperature (RT) for 2 h. The IsdB binding was detected using Avidin-IgG conjugated to Horseradish peroxidase (HRP) and the Tetramethylbenzidine (TMB) substrate, and the optical density (OD)_450_ was measured using a microplate reader. The bar graph displays the OD values corresponding to the 1:10 molar IsdB:antibody ratio. *n* = 3. (**h**) Human peripheral blood mononuclear cells (PBMCs) were left untreated or stimulated with IsdB (10 µg/mL) or IsdB preincubated with increasing molar ratios of a human monoclonal anti-IsdB antibody for 6 h. Data are represented as mean ± SEM of indicated biological replicates (“*n*”) performed in technical duplicate or triplicate. Each point represents one donor (human monocytes) or biological replicate (mBMDCs). One-way ANOVA (Fig. 2g) or two-way ANOVA (Fig. 2a and b) was utilized to determine statistical significance. *P < 0.05, **P < 0.01, ***P < 0.001, and ****P < 0.0001 represent IsdB or LPS vs untreated cells. *^##^P* < 0.01 IsdB vs IsdB + inhibitor (S). *^$$^P* < 0.01 and *^$$$$^P* < 0.0001 represent LPS vs LPS + inhibitor (S). CLI-095, TLR4 inhibitor; Fnorm, normalized fluorescence; PK, proteinase K. ϕ represents respective controls or unstimulated cells.

Next, we examined the mechanism(s) of the IsdB-induced cytokine release. The LPS sensor TLR4 also recognizes several *S. aureus* toxins (9, 10). When we pre-incubated human monocytes or mBMDCs with the TLR4 signaling inhibitor CLI-095, the IL-6 release in response to IsdB and Lac-IsdB was abolished as was the release of all other cytokines (Fig. 2a and b; Fig. S4). The response to LPS was also significantly reduced, underlining the potency of the inhibitor. Likewise, IsdB-induced release of IL-6 was strongly inhibited in the presence of an anti-TLR4 antibody ( Fig. S4a). Furthermore, mBMDCs from TLR4- or MyD88-KO mice failed to secrete IL-6 when stimulated with IsdB (Fig. 2c). Taken together, these data demonstrate that IsdB induces IL-6 release in a TLR4-dependent manner.

### IsdB binds to TLR4

To elucidate the molecular details of the IsdB-TLR4 interaction, we performed molecular docking. We generated 16-model complexes of hTLR4 and IsdB, performed electrostatic calculations, and evaluated their electrostatic compatibility in complex formation. The results predicted that IsdB binds directly to human TLR4 (Fig. 2d). Based on this analysis, six models were selected for further analysis by DimpPlot to visualize the hydrophobic and non-covalent interactions. Dim plot analysis identified the amino acids valine (V)-189 and tyrosine (Y)-192 in the NEAT1 domain of IsdB as partners in hydrophobic interaction with glutamine (Q)-188 and serine (S)-123 of the modeled TLR4 complex, respectively (Fig. 2e). In addition, glutamic acid (E) residues, present at positions 326, 329, and 332 in the linker between the NEAT1 and NEAT2 domains of IsdB, form salt bridges with lysine (K) residues of TLR4. Likewise, lysine (K)-321 in the linker of IsdB forms a salt bridge with glutamic acid (E) on TLR4 (not shown). Together, these interactions represent strong non-covalent binding between IsdB and TLR4.

We next performed MST to measure the binding strength between IsdB and TLR4. To this end, the recombinant human TLR4 (rhTLR4) was labeled with an NHS-ester dye and incubated with increasing concentrations of recombinant IsdB (*E. coli*-derived IsdB and Lac-IsdB). This confirmed the predicted high-affinity binding of IsdB and Lac-IsdB to rhTLR4 with Kds of 98.3 and 150 nM, respectively (Fig. 2f). LPS, the prototypic TLR4 ligand, failed to show significant interaction with rhTLR4 (Kd: 0.4 mM) as it requires the presence of myeloid differentiation factor 2 (MD2) for binding to TLR4 and TLR4 dimerization (38) (Fig. 2f). The fact that IsdB binds strongly to TLR4 under conditions that do not allow LPS binding—in the absence of MD2—strongly supports the *in silico* prediction of a direct interaction between the two molecules.

### An anti-IsdB antibody significantly blocks the binding of IsdB to TLR4

In view of the high affinity of IsdB for recombinant TLR4, we investigated whether antibodies targeting IsdB could hinder this binding. To address this, we performed a solid-phase binding assay (ELISA), using polyclonal rabbit antibodies against IsdB. First, we incubated TLR4 with increasing concentrations of recombinant IsdB, ranging from 1 to 20 µg/mL. IsdB showed concentration-dependent binding to TLR4 (Fig. S5). Notably, the highest binding occurred at an IsdB concentration of 2 µg/mL. Therefore, we chose an IsdB concentration of 1 µg/mL for subsequent experiments. Next, we incubated IsdB with or without increasing molar ratios of anti-IsdB antibodies. For control, we used a mouse anti-IgG antibody. Indeed, at a molar ratio of 10:1, anti-IsdB antibodies significantly hindered the binding of IsdB to TLR4 compared with the control antibodies (Fig. 2g).

To further investigate whether anti-IsdB antibodies could also neutralize the IsdB-induced release of IL-6, we developed a human anti-IsdB monoclonal antibody (IsdB-mAb). We treated PBMCs with IsdB or IsdB pre-incubated with increasing molar ratios of the IsdB-mAb for 6 h, and IL-6 release was determined by ELISA. As shown in Fig. 2h, preincubation of IsdB with the IsdB-mAb at a molar ratio of 1:1 (IsdB:mAb) strongly reduced the IsdB-induced IL-6 release by human PBMCs. At an IsdB:mAb ratio of 1:4, the IL-6 release was completely abolished. Together, these results show that anti-IsdB antibodies can block the binding of IsdB to TLR4, reinforcing our finding that IsdB forms a binding interaction with TLR4.

### IsdB acts via the TLR4-MyD88-NF-κB signaling pathway

**Fig 3:**
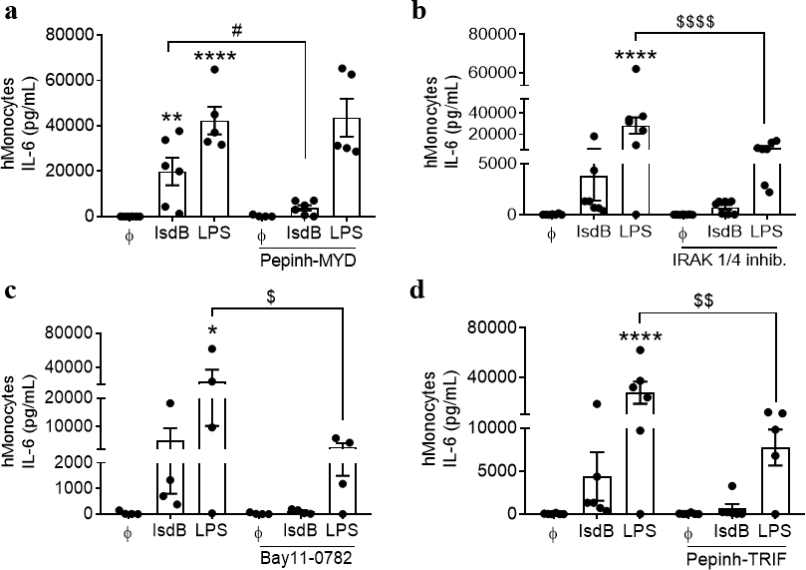
IsdB induces proinflammatory cytokine release via the MyD88-NF-κB signaling cascade (**a–d**) Human monocytes were left untreated or pre-treated with IRAK1/4 inhibitor (1 µM) (**b**) or Bay11-0782 (10 µM) (**c**) for 45 min or with Pepinh-MYD (50 µM) (**a**) or Pepinh-TRIF (50 µM) (**d**) for 6 h before treatment with either IsdB (10 µg/mL) or LPS (100 ng/mL) for additional 24 h. *n* = 6 for a, b, and d; *n* = 4 for c. Cell-free supernatants were analyzed for the release of IL-6 by ELISA. Data are represented as mean ± SEM of indicated biological replicates (“*n*”) performed in technical duplicate or triplicate. Each point represents one donor. Two-way ANOVA was utilized to determine statistical significance. *P < 0.05, **P < 0.01, and ****P < 0.0001 represent IsdB or LPS vs untreated cells. *^#^P* < 0.05 represents IsdB vs IsdB + inhibitor (**S**). *^$^P* < 0.05, *^$$^P* < 0.01, and *^$$$$^P* < 0.0001 represent LPS vs LPS + inhibitor (**S**). Bay11-0782, NF-κB inhibitor; IRAK1/4 inhib., IRAK1/4 inhibitor; Pepinh-MYD, MyD88 inhibitor; Pepinh-TRIF, TRIF inhibitor. ϕ represents respective controls or unstimulated cells.

Next, we explored the IsdB-induced signaling pathways downstream of TLR4. TLR4 can signal through two adaptor molecules, the myeloid differentiation primary response 88 (MyD88) and the TIR-domain-containing adapter-inducing interferon-β (TRIF), leading to NF-κB-dependent induction of proinflammatory cytokines (39–41). Inhibition of MyD88 in human monocytes with Pepinh-MYD (Fig. 3a) or genetic deletion of MyD88 in mBMDCs (Fig. 2c) abolished the IL-6 release in response to IsdB and Lac-IsdB. Pre-incubation of cells with an IRAK1/4 inhibitor (protein kinase involved in the MyD88-dependent signaling) or the NF-κB inhibitor Bay11-0782 before IsdB treatment also dramatically reduced the IL-6 release (Fig. 3b and c). In contrast, blockade of MyD88 did not affect the response to LPS (Fig. 3a), suggesting that monocytes may use another pathway for LPS signaling, as previously reported (42). This difference in the mechanisms of action between the two bacterial factors corroborates the view that IsdB binds to and activates TLR4 directly, independent of LPS.

To investigate the influence of TRIF on IL-6 release via NF-κB, we incubated human monocytes with the TRIF inhibitor Pepinh-TRIF before treating them with IsdB or LPS (Fig. 3d). Blockade of TRIF significantly reduced the IsdB- and LPS-induced IL-6 release (Fig. 3d), indicating that TRIF contributes to priming of the NF-κB in response to LPS as reported (39). These observations demonstrate that IsdB induces proinflammatory cytokines via the TLR4-MyD88-IRAK1/4/TRIF-NF-κB signaling pathway.

### IsdB activates the NLRP3-Caspase-1 inflammasome and induces the release of IL-1β

**Fig 4:**
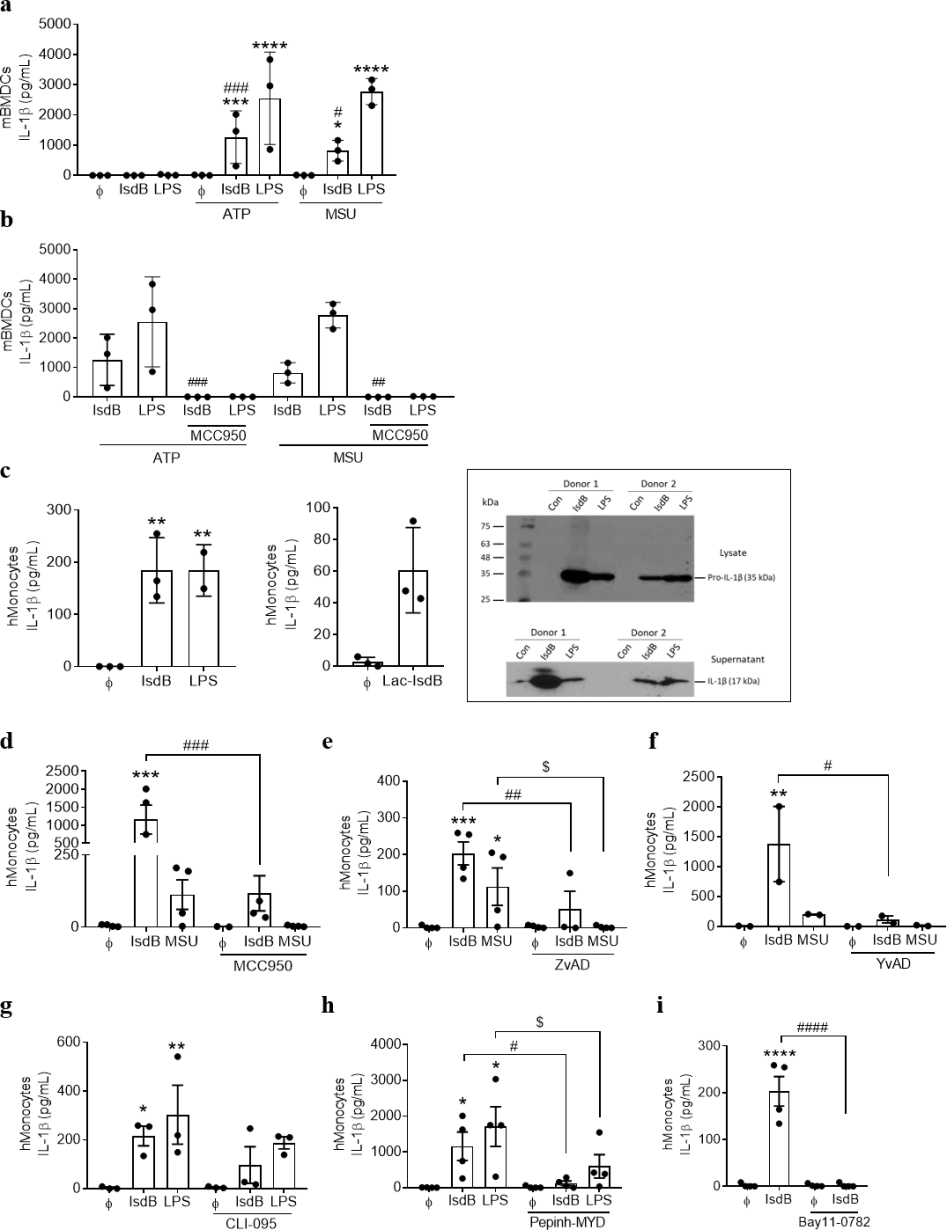
IsdB activates the NLRP3 inflammasome to generate IL-1β. (**a**) mBMDCs were left unprimed or primed with either IsdB (10 µg/mL) or LPS (1 ng/mL) for 3 h followed by treatment with ATP (5 mM) or MSU (200 µg/mL) for additional 6 h, *n* = 3. (**b**) IsdB- or LPS-primed mBMDCs were incubated with or without MCC950 (5 µM) for 45 min followed by treatment with ATP (5 mM) or MSU (200 µg/mL) for additional 6 h, *n* = 3. Cell-free supernatants in a and b were analyzed for mouse IL-1β release by ELISA. (**c**) Human monocytes were left unstimulated or stimulated with IsdB (10 µg/mL) or Lac-IsdB (10 µg/mL) or LPS (100 ng/mL) for 24 h, *n* = 3. The amount of secreted IL-1β in cell culture supernatants was measured by ELISA or visualized by western blot, and the pro-IL-1β in cell lysates was detected by western blot. (**d–i**) Human monocytes were left untreated or pre-treated with the indicated signaling inhibitors for 45 min except for Pepinh-MYD (6 h) (**h**) prior to treatment with IsdB (10 µg/mL) for an additional 24 h. MCC950 (5 µM) *n* = 4, ZvAD (20 µM) *n* = 4, YvAD (30 µg/ml) *n* = 2, CLI-095 (1 µM) *n* = 3, Pepinh-MYD (50 µM) *n* = 4, or Bay11-0782 (10 µM) *n* = 4. In experiments d–f, MSU (200 µg/mL) served as a positive control. In experiments g–h, LPS (100 ng/mL) served as a positive control. IL-1β levels in the supernatants were determined by ELISA. Data are displayed as mean ± SEM of indicated biological replicates (“*n*”) performed in technical duplicate or triplicate. Each point represents one donor (human monocytes) or biological replicate (mBMDCs). In Fig. 4c, one-way ANOVA (IsdB) or paired *t*-test (Lac-IsdB) was utilized to compare the statistics between the groups. Two-way ANOVA was utilized to determine statistical significance between the groups in Fig. 3d through h. *P < 0.05, **P < 0.01, ***P < 0.001, and ****P < 0.0001 represent IsdB or LPS or MSU vs untreated cells or respective controls. *^#^P* < 0.05, *^##^P* < 0.01, *^###^P* < 0.001, and *^####^P* < 0.0001 represent IsdB vs IsdB + inhibitor (S), IsdB vs IsdB + ATP, or MSU. *^$^P* < 0.05 represents MSU vs MSU + inhibitor (S). ATP, adenosine triphosphate; Bay11-0782, NF-κB inhibitor; CLI-095, TLR4 inhibitor; MCC950, NLRP3 inhibitor; MSU, monosodium urate; Pepin-MYD, MyD88 inhibitor. ϕ represents respective controls or unstimulated cells.

IsdB also induced the secretion of IL-1β (Fig. 1d). However, while activation of the TLR4-NF-κB-pathway is sufficient for the release of IL-6 and many other inflammatory cytokines, the release of mature IL-1β requires the activation of the inflammasome in addition. We therefore studied the inflammasome pathway in detail. Inflammasomes are multimeric protein complexes, which act as crucial mediators of the innate immune response, fulfilling an essential role in bacterial clearance and inflammation. The NLRP3 inflammasome is the best characterized. Its activation and the release of IL-1β usually require two signals. The priming signal by the TLR pathway leads to the production of pro-IL-1β, and the second signal—provided by various danger signals such as ion flux, extracellular adenosine 5′-triphosphate (ATP) or reactive oxygen species (ROS)—induces the caspase-1-dependent cleavage of pro-IL-1β to IL-1β, the mature form of the cytokine (43–45). We prepared mBMDCs and stimulated them with IsdB or LPS for 3 h before adding ATP or monosodium urate crystals (MSU) for another 6 h. IL-1β release in cell-free supernatants was assessed by ELISA. As expected in mBMDCs, priming with IsdB or LPS alone did not result in IL-1β secretion, but the cells required an additional activation signal such as extracellular ATP or MSU (Fig. 4a). Inhibition of NLRP3 by the specific inhibitor MCC950 abolished the IL-1β release (Fig. 4b). Adding IsdB to LPS-primed mBMDCs did not induce IL-1β production, corroborating the notion that both act as priming signals via TLR4 (Fig. S6). These findings demonstrate that in mBMDCs, IsdB binding is the first step in the two-step activation of the NLRP3 inflammasome for the release of IL-1β.

Human monocytes regulate the release of IL-1β differently than mBMDCs (42). We found that IsdB or Lac-IsdB alone, without additional stimulus, induced a notable production of pro- and active forms of IL-1β in human monocytes (Fig. 4c). The same was true for LPS (Fig. 4c) and MSU (Fig. 4d through f), which served as positive controls. All responses were abolished by NLRP3 inhibition with MCC950 (Fig. 4d). Moreover, similar to NLRP3 inhibition, the pan-caspase inhibitor ZvAD and the specific caspase-1 inhibitor YvAD also significantly reduced the IL-1β production in human monocytes (Fig. 4e and f). Inhibition of TLR4, MyD88, or NF-κB with CLI-095, Pepinh-MYD, or Bay11-0782, respectively, also abrogated the IL-1β production in human monocytes (Fig. 4g through i). Thus, in human monocytes, both the TLR4-pathway and activation of the NLRP3 inflammasome are necessary to generate mature IL-1β.

**Fig 5:**
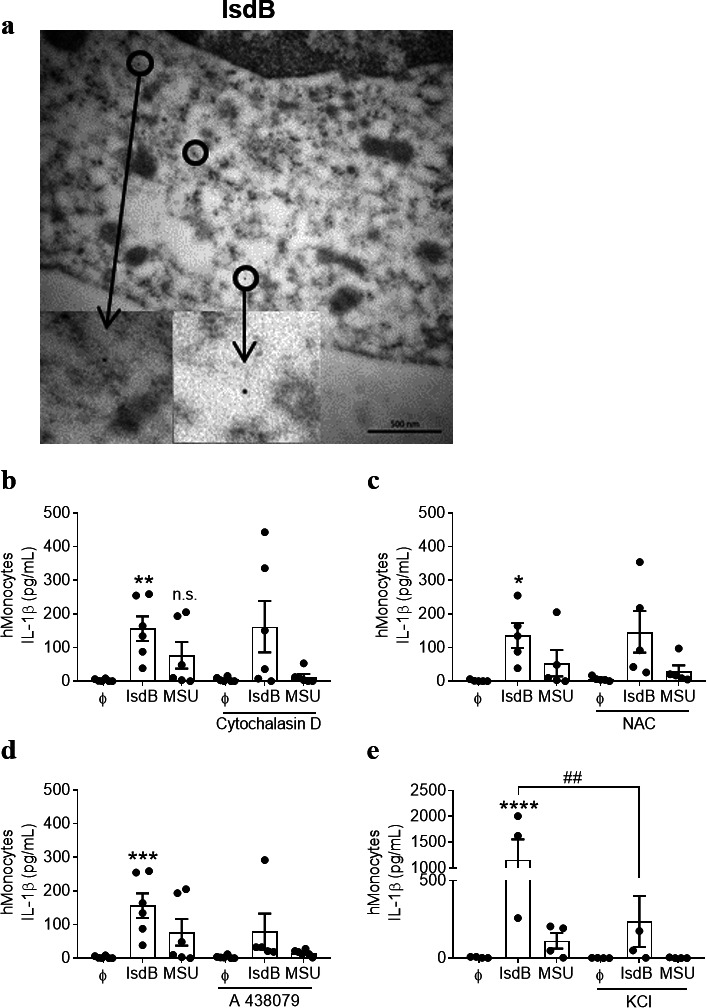
Molecular mechanisms of IsdB-induced IL-1β release. (**a**) Human monocytes were treated with IsdB (10 µg/mL) for 24 h. Cells were fixed and subjected to immunogold labeling. Transmission electron micrographs show the localization of IsdB in the cytoplasm. A representative image of one experiment is shown. Scale bar = 500 nm. (**b**) Human monocytes were incubated in the presence or absence of Cytochalasin D, 5 µM (**b**); NAC, 10 mM (**c**); A438079, 100 µM (**d**); or KCl, 75 mM (**e**) for 45 min followed by treatment with IsdB (10 µg/mL) or MSU (200 µg/mL) for additional 24 h. Cell-free supernatants were analyzed for IL-1β by ELISA. *n* = 6 in b–d; *n* = 3 in e. Data are the mean ± SEM of indicated biological replicates (“*n*”) performed in technical duplicate to triplicate. Each point represents one donor. Two-way ANOVA was utilized to determine statistical significance. *P < 0.05, **P < 0.01, ***P < 0.001, and ***P < 0.0001 represent IsdB or MSU vs untreated cells. *^##^P* < 0.01 represents IsdB vs IsdB + KCl. A438079, P2X7 inhibitor; Cytochalasin D, phagocytosis inhibitor; MSU, monosodium urate; NAC, N-acetyl cysteine; n.s., non-significant. ϕ represents respective controls or unstimulated cells.

The NLRP3 inflammasome can also be activated by the phagocytic uptake of crystals (e.g., MSU, silica, and alum), nanoparticles, or β-amyloid peptides that cause rupture of the phagolysosome and release of ROS (46–48). Transmission electron microscopy (TEM) of IsdB-treated monocytes revealed that some IsdB was localized in the cytosol, as evident by immunogold staining (Fig. 5a). However, blockade of phagocytosis with cytochalasin D (CytD) or neutralization of ROS with the scavenger N-acetyl cysteine (NAC) did not interfere with the IsdB-induced production of IL-1β (Fig. 5b and c). In response to MSU crystals, however, both CytD- and ROS inhibitors greatly reduced the generation of IL-1β as expected (Fig. 5b and c).

The purinergic receptor P2X7 and potassium efflux contribute to MSU-induced IL-1β release (49). Inhibition of P2X7 with the antagonist A438079 trended to reduce the IL-1β release in response to IsdB and blocking potassium (K^+^)-efflux by altering the extracellular ion homeostasis with potassium chloride (KCl) significantly reduced IL-1β release (Fig. 5d and e). Both manipulations almost abolished the MSU-induced IL-1β release (Fig. 5d), indicating that the IsdB- as well as the MSU-induced IL-1β production in monocytes involve (i) activation of P2X7 receptor and autocrine secretion of ATP and (ii) K^+^-efflux. Thus, purinergic receptors contribute to the activation of the inflammasome by IsdB, while ROS and/or phagocytosis are not involved which is, hence, different from the mode of activation reported for MSU (48, 50).

### Deletion of IsdB reduces the IL-1β-release by *S. aureus*-infected human monocytes

**Fig 6:**
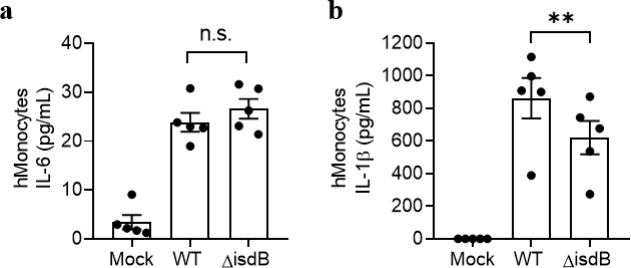
Deletion of *isdB* reduces the IL-1β-release by *S. aureus*-infected human monocytes. Human monocytes were untouched or infected at an multiplicity of infection (MOI) of 25 with wild-type *S. aureus* Newman (WT) or an *isdB*-deficient mutant strain *(*Δ*isdB*) for 1 h. (**a**) IL-6 and (**b**) IL-1β release was measured in the supernatants by a bead-based multiplex assay, *n* = 5. Data are the mean ± SEM of indicated biological replicates (“*n*”) performed in technical duplicate to triplicate. Each point represents one donor. One-way ANOVA was utilized to determine statistical significance between the groups. **P < 0.01 represents WT vs. *ΔisdB*. n.s., non-significant. Mock represents unstimulated cells.

For a closer view on the significance of TLR4/IsdB interaction *in vitro*, we infected monocytes with wild-type or isogenic *isdB*-deficient (Δ*isdB*) *S. aureus* Newman strains and compared cytokine production. Infection with live wild-type *S. aureus* induced the release of IL-6 and IL-1β. The lack of IsdB did not affect IL-6 secretion (Fig. 6a) but significantly reduced the release of IL-1β (Fig. 6b). This shows that other virulence factors can compensate for IsdB in the induction of IL-6, whereas IsdB is necessary for maximal IL-1β release.

## DISCUSSION

We have discovered that the iron scavenger IsdB of *S. aureus* is sensed as a PAMP by immune cells. IsdB binds directly to TLR4, leading to activation of the NLRP3 inflammasome and the release of IL-1β. This identifies yet another role of IsdB in host-pathogen interaction besides bacterial iron acquisition and adhesion of *S. aureus* to host cells (31–35).

Since TLR4 is a very sensitive receptor for LPS, it was crucial to exclude activation by contaminating LPS. We did this by (i) rigorously depleting LPS from the IsdB preparations generated in *E. coli*, (ii) producing Lac-IsdB in *Lactococcus lactis*, a Gram-positive bacterium lacking LPS, and (iii) demonstrating that IsdB binds to TLR4 with high affinity under conditions where LPS is unable to do so. The recombinant Lac-IsdB had the same properties as the *E. coli*-derived IsdB preparations in all experiments, and microscale thermophoresis revealed high-affinity binding of recombinant IsdB to recombinant TLR4 in the absence of MD-2, which is strictly required for the interaction of LPS with TLR4.

Other possible confounders of concern are Hb and heme. During infection with *S. aureus*, they can be released from erythrocytes after lysis by the pore-forming toxins Hla and LukED (19–21). Both are ligands of IsdB; the NEAT1 domain has high affinity for Hb, while the NEAT2 domain is involved in the extraction of the heme group from Hb (26, 28). Moreover, Hb and heme can bind to TLR4, thereby acting as danger-associated molecular patterns for immune cells. However, our recombinant IsdB and TLR4 preparations were generated in Hb- and heme-free media. Still, the two proteins interacted with high affinity in the microscale thermophoresis experiments. This shows that the strong binding of IsdB to TLR4 is independent of Hb or heme. In line with this, *in silico* molecular docking predicted that IsdB binds directly to human TLR4. DimPlot analysis identified the glutamine and serine residues in TLR4 that can form hydrophobic interactions with valine189 and tyrosine192 in the IsdB NEAT1 domain, and lysine residues in TLR4 that can form salt bridges with three glutamic acid residues in the linker between NEAT1 and NEAT2. These are clearly distinct from the heme- and Hb-binding motifs of IsdB, ^163^QFYHYAS^169^ in the NEAT1, and ^440^YDGQY^444^ in the NEAT2 domain, respectively (27, 51). Future site-directed mutagenesis experiments are required to validate these predictions. Altering valine189 and tyrosine192 in the NEAT1 domain to aspartic acid and histidine, respectively, as well as converting glutamic acid residues within the linker domain to lysins, are expected to reduce the binding affinity of IsdB for TLR4.

The interaction between IsdB and TLR4 was of high affinity with a mean Kd of 98 nM. Still, anti-IsdB antibodies interfered with binding to TLR4 (Fig. 2g) and abolished the production of IL-6 (Fig. 2h). Other known TLR4-binding virulence factors of *S. aureus*, the phenol soluble modulins PSMα1, PSMα2, PSMα3, PSMβ1, and PSMβ2, bind to TLR4 with at least 30-fold lower affinity, their Kds ranging from 3.0 to 7.8 µM (10).

TLR4 was necessary for the IsdB-mediated cytokine induction in human monocytes and murine BMDCs because the effect was abrogated by TLR4 knockout and by the small molecule TLR4 inhibitor CLI-095. TLR4 can signal through the adaptor proteins MyD88 and TRIF (12, 52–54). Both signaling pathways lead to the activation of the transcription factor NF-κB and the production of proinflammatory cytokines. Inhibition of MyD88 showed that IsdB but not LPS uses this pathway to induce IL-6 production in human monocytes. This is in agreement with reports that LPS signals through TRIF (42). MyD88 and TRIF dual signaling is required for the activation of the interferon-responsive factor (IRF)-3 that promotes the release of the type 1 interferons IFNα, IFNβ, and IFNλ (39). Since IsdB elicited hardly any IFNα2 (2–6 pg/mL), it does not strongly induce the IRF-3 pathway (Fig. S3), corroborating that the release of proinflammatory cytokines by IsdB is mediated predominantly by the TLR4-MyD88-NF-κB axis.

The TLR4-MyD88-NF-κB axis controls two distinct but connected processes: metabolic rewiring and trained immunity/trained tolerance (55–58). LPS binding to TLR4 dramatically alters the cellular metabolism. The cells enhance glucose uptake, glycolysis, and the tricarboxylic acid cycle to produce effector molecules such as IL-6 and IL-1β, while minimizing oxidative phosphorylation (59). This is known as the Warburg effect (60). Since IsdB activates the same signaling axis, it is plausible that IsdB could also rewire the metabolism in innate immune cells, thereby promoting proinflammatory gene expression. After initially mounting a strong inflammatory response, LPS-stimulated innate immune cells acquire an innate form of memory. This phenomenon is known as LPS-induced innate tolerance (also referred to as LPS tolerance), an epigenetic and metabolic state in which the cells’ inflammatory response to secondary stimuli is severely compromised (57, 61, 62). It is tempting to speculate that by triggering the TLR4/MyD88 axis, IsdB may induce a similar immunosuppressive effect, which would probably benefit *S. aureus*. However, this remains to be investigated.

IsdB stimulated human monocytes and mBMDCs to secrete large amounts of IL-1β, which was dependent on the NLRP3-caspase-1 inflammasome. The canonical inflammasome pathway requires a priming signal for the synthesis of pro-IL-1β and a second “danger” signal to activate caspase-1. Then, pro-IL-1β is cleaved to generate the mature, functional IL-1β. Since IsdB also stimulated the production of other inflammatory cytokines like IL-6 through TLR4, we assumed that the bacterial virulence factor provides the priming signal. This was the case: in mBMDCs, the production of IL-1β in response to IsdB (or LPS) required an additional danger signal, ATP or MSU. In contrast, IsdB on its own was able to trigger the release of IL-1β in human monocytes. These cells use an alternative pathway of NLRP3 inflammasome activation as they constitutively express active caspase-1 due to endogenous ATP release. Hence, a single signal, LPS or MSU—and now IsdB—suffices to trigger the release of the mature IL-1β in human monocytes (42).

*S. aureus* has several means to activate the NLRP3 inflammasome and induce IL-1β (14, 63–66). The staphylococcal superantigen TSST-1 can act as the priming signal. In mouse peritoneal macrophages, the toxin induces TLR4- and NLRP3-dependent IL-1β production in the presence of ATP (67). In contrast, *S. aureus* α-, β-, and γ-hemolysins act as the second signal. They induce the release of IL-1β via NLRP3 and caspase-1 only after the cells have been primed, e.g., with lipoproteins via TLR2 (66, 68). Thus, *S. aureus* can fully activate the canonical NLRP3 inflammasome pathway in immune cells and induce mature IL-1β: IsdB, lipoproteins, or TSST-1 can prime, while staphylococcal hemolysins may provide the second signal. ATP leaking from dying host cells or ROS generated during oxidative burst in immune cells could also act as the second signal.

*S. aureus* virulence is characterized by marked redundancy. To assess the importance of IsdB in anti-*S. aureus* immune defenses, we infected monocytes with live WT *S. aureus* Newman and an isogenic *isdB* deletion mutant. The IsdB defect did not affect IL-6 secretion. Here, IsdB is redundant and can be fully compensated by other virulence factors of *S. aureus* Newman. In contrast, IsdB was necessary for maximal IL-1β release. This allows two conclusions: (i) IsdB has an essential role in the activation of the inflammasome by live *S. aureus* bacteria and (ii) in *S. aureus* infection, IsdB significantly contributes to the activation of innate defense mechanisms.

The NLRP3 inflammasome and IL-1β influence the course of *S. aureus* infection in mouse models. IL-1β-derived neutrophils are essential for abscess formation and clearance of *S. aureus* infection. The net effect of IL-1β, however, bacterial invasion or immune control, depends on the context (65). For instance, mice deficient for NLRP3 or caspase-1 are protected from *S. aureus* pneumonia (63, 69).

In the past, IsdB was studied as an *S. aureus* vaccine candidate for two reasons: it is an immunodominant bacterial antigen that induces a robust antibody response in humans and in mice (36, 37, 70–74), and its iron scavenger function is essential for *S. aureus* survival in the host. Vaccination with IsdB without adjuvant (V710) strongly boosted the specific antibody response (71). Yet, in a phase IIb/III randomized placebo-controlled clinical trial, the vaccine did not protect from *S. aureus* infection. Among patients who developed surgical site infections, a higher mortality was associated with the use of the vaccine (72, 75, 76). In search of an explanation, researchers have suggested several mechanisms, which are mutually non-exclusive: (i) many affected patients had strikingly low serum concentrations of inflammatory cytokines, especially IL-2 and IL-17A, prior to vaccination, which may have put them at increased risk (72), (ii) anti-IsdB antibodies favored systemic dissemination of *S. aureus* in a mouse model of surgical site infection, if the antibodies did not block Hb binding to IsdB (77), and (iii) in *S. aureus*-naïve mice, IsdB vaccines were protective, while infection with *S. aureus* elicited non-protective IsdB-specific antibodies. Vaccination of previously infected mice recalled and boosted the non-protective antibody response to IsdB, which suppressed vaccine-induced protection (78). Our results suggest yet another mechanism: we propose that antibodies that block the binding of IsdB-NEAT1 to TLR4 may attenuate the innate immune response to *S. aureus* infection and weaken the anti-bacterial defense. Our findings that (i) blocking antibodies abolished IsdB-mediated cytokine induction in human monocytes and (ii) human monocytes produced less IL-1β after infection with *isdB*-deficient live *S. aureus* than with isogenic WT bacteria lend support to this notion.

In conclusion, we have discovered a new role for IsdB in *S. aureus*-host interaction, which appears to be significant for the defense against infection with this pathogen.

## MATERIALS AND METHODS

Anti-TLR4 antibody, ATP, Bay 11-0782 (NF-κB signaling inhibitor), CLI-095 (TLR4 inhibitor), Cytochalasin D (phagocytosis inhibitor), LPS-EB ultrapure (*E. coli* O111:B4B), MCC950 (NLRP3 inhibitor), MSU, Pepinh-MyD88 (MyD88-inhibitory peptide), Pepinh-TRIF (TRIF inhibitor), YvAD (caspase-1 inhibitor), and ZvAD (pan-caspase inhibitor) were purchased from InvivoGen (San Diego, USA). A438079 (P2X7 inhibitor) and IRAK1/4 inhibitor were obtained from Tocris (Bristol, UK). β-Mercaptoethanol (β-ME), N-acetyl cysteine (ROS inhibitor), and poly-L-lysine, Amicon Ultra-0.5 Centrifugal Filter Units (10 kDa cutoff) were obtained from Sigma-Aldrich (St. Louis, Missouri, USA). Human Pancoll solution, human pooled serum, non-essential amino acids (NAA), phosphate-buffered saline (PBS), Roswell Park Memorial Institute (RPMI) media, and sodium pyruvate were purchased from PAN-Biotech (Aidenbach, Germany). Penicillin, streptomycin, and glutamine were obtained from Gibco (Waltham, Massachusetts, USA). CD14 microbeads were obtained from Miltenyi Biotech (Bergisch Gladbach, Germany) or STEMCELL Technologies (Cambridge, MA, USA) and murine granulocyte-macrophage colony-stimulating factor (GM-CSF) from PeproTech (Cranbury, USA). Human TLR4 recombinant active proteins (rhTLR4) were purchased from Abcam (Cambridge, UK). TMB substrate was from BD Biosciences (California, USA). All cell culture material was either purchased from Thermo Scientific or Nuclon (Thermo Fisher, Waltham, Massachusetts, USA). All ELISA kits (ELISA MAX Deluxe Set) and the multiplex LEGENDplex Human Inflammation Panel 1, 13-plex kit were purchased from BioLegend, San Diego, USA. The human Cytokine/Chemokine/Growth Factor Panel A (Milliplex_MAP_) kit was obtained from Millipore Sigma-Aldrich. The Cytotoxicity Detection Kit Lactate Dehydrogenase (LDH) was purchased from Roche Diagnostics/Sigma-Aldrich. The monoclonal anti-His_5_-antibody was obtained from Qiagen (Venlo, The Netherlands). Rabbit polyclonal IL-1β antibodies were obtained from Cell Signaling, (#83186) and Santa Cruz Biotechnology Inc. (#sc-7884). The anti β-actin antibody and goat anti-rabbit IgG conjugated to HRP (#7074S) were obtained from Santa Cruz Technology.

### Expression and purification of His_6_-tagged recombinant IsdB proteins

#### Expression of His_6_-tagged IsdB in *E. coli*

*E. coli* strain SCS1 (San Diego, California, USA) was obtained from the Department of Functional Genomics, Interfaculty Institute for Genetics and Functional Genomics, University Medicine Greifswald. *E. coli* SCS1 cells were transformed with plasmid pQE30/pSE111 (Qiagen, Maryland, USA) encoding His_6_-tagged IsdB (hereafter referred to as IsdB) and were grown to OD_595_ of 0.5 at 37°C in LB medium (Sigma-Aldrich) supplemented with 100 µg/mL of ampicillin and 30 µg/mL of kanamycin. Overexpression was induced with 1 mM IPTG (Sigma-Aldrich) for 3 h, and the bacteria were collected by centrifugation and resuspended in binding buffer (0.5 M NaCl, 20 mM Na_2_HPO_3_, and 30 mM Imidazole) supplemented with 1% Triton X-100. Aliquots of pre- and post-IPTG bacterial cultures were subjected to SDS-PAGE to confirm the overexpression.

#### Expression of His_6_-tagged IsdB in *Lactococcus lactis*

IsdB was also expressed in *Lactococcus lactis* PA1001 (hereafter referred to as Lac-IsdB) using plasmid pNG4110-isdB as previously described (79).

#### Purification of His_6_-tagged recombinant IsdB proteins

IsdB in ultra-filtered *E. coli-*bacterial lysates or Lac-IsdB in the growth medium fraction were purified by affinity chromatography using Ni^2+^ sepharose columns (His-trap HP 1 mL column, Amersham Biosciences/Cytiva) and an ÄKTA-FPLC system (GE Healthcare, Chalfont St Giles, UK). The proteins were eluted from the columns using the elution buffer (20 mM sodium phosphate buffer pH 7.4, 0.5 M NaCl, and 500 mM imidazole), and subsequently, the elution buffer was exchanged for PBS either by ultrafiltration (IsdB) (Millipore, Burlington, Massachusetts, USA) or dialysis (Lac-IsdB). In the case of IsdB, LPS depletion was performed with an EndoTrap RED Kit (Lionex, Braunschweig, Germany), and the endotoxin concentration was determined by using LAL-based Endosafe PTS cartridges (Jackson Laboratories, Bar Harbor, California, USA).

### Cell culture

Buffy coats or Leukopaks were obtained from anonymous blood donors with informed consent from the University Medicine Greifswald and New York Blood Center, respectively. Human PBMCs in buffy coat preparations or Leukopaks were isolated using the Ficoll gradient method as previously described (80). Monocytes were isolated by the plate-adherent method or using CD14 microbeads according to the manufacturer’s instructions. The human monocytes were cultured at a density of 0.5 × 10^6^/mL (unless mentioned) and stimulated in RPMI media supplemented with 5% heat-inactivated human pooled serum, 1% penicillin, streptomycin, glutamine (PSG), 1% sodium pyruvate, 1% NAA, and 50 µM β-ME (hereafter referred to as complete medium).

mBMDCs were prepared using an established protocol. Briefly, bone marrow cells from 6- to 8 week-old WT, TLR4-, or MyD88-KO mice (all mice on C57BL/6 background) were differentiated into mBMDCs using GM-CSF (10 ng/mL) for 7 days. Cells were cultured in RPMI medium supplemented with 10% fetal calf serum, 1% penicillin and streptomycin, glutamine, and 50 µM β-ME. Cells were fed with fresh medium containing GM-CSF at days 3 and 6. After 7 days, the non-adherent cells were collected and cultured at a density of 1 × 10^6^/mL in 12- or 24-well plates overnight before use. All mBMDCs were stimulated in serum-free medium.

### *S. aureus* infection experiments

*S. aureus* Newman WT (VJT 1.01) and Newman Δ*isdB* (VJT 1.03) were streaked on TSA plates and grown overnight at 37°C. The following evening, overnight cultures were prepared in RPMI supplemented with 1% casamino acids and 200 µM dipyridyl. The bacterial strains were centrifuged, washed with 5 mL of PBS, and subsequently resuspended in PBS. The strains were normalized to an optical density at 600 nm of 1 and diluted to achieve a MOI of 25 upon addition to monocytes.

Monocytes were resuspended in clear RPMI medium supplemented with 10% fetal bovine serum and plated in 96-well tissue culture plates at a density of 2 × 10^5^/well. The wells were then infected with bacteria, and the infection was synchronized by centrifuging the plates at 290 *× g* for 5 min. The infection was then carried out for 1 h at 37°C, 5% CO_2_. After 1 h, plates were spun at 453 *× g* for 5 min. The cell-free supernatants were harvested and stored at −20°C until further use.

### ELISA and multiplex assay for cytokine measurement

All measurements were performed according to manufacturer’s instructions. The concentration of human or mouse IL-6 and IL-1β in the cell-free culture supernatants was determined by ELISA using a TMB substrate. The absorbance was measured at OD_450_ on an ELISA reader (Tecan Infinite M200, Tecan Group AG, Männedorf, Switzerland). In some cases, supernatants were subjected to an extensive cytokine analysis, including the measurement of CCL2, IFNα2, IL-1β, IL-12p70, IL-23, IL-33, and TNFα. This was done using bead-based multiplex assays with a human proinflammatory cytokine multiplex kit (LegendPlex) or human Cytokine/Chemokine/Growth Factor Panel A kit (Milliplex_MAP_). The samples were analyzed on an LSRII instrument (BD Biosciences) or on a MAGPIX system.

### Western blot

Cell-free supernatants were concentrated using Amicon filters, following the manufacturer’s instructions. Cells were lysed in buffer containing TRIzol, and proteins were precipitated with TRIzol and isopropanol. Protein concentration was determined using the Bradford method. Subsequently, 20 µg of protein was separated by SDS-PAGE and transferred to a polyvinylidene fluoride membrane (Immobilon-P, Merck.) After blocking the membrane for 1 h at RT with 5% non-fat milk in TBST, it was incubated overnight with antibodies against IL-1β (1:1,000) or β-actin (1:2,000). Following this, the membrane was exposed to appropriate secondary antibodies, and signals were detected using a chemiluminescence substrate (SuperSignal West Femto Maximum Sensitivity Substrate, Thermo Scientific).

### TEM and immunogold labeling

Isolated human monocytes were cultured at a concentration of 2 × 10^6^/mL in 6-well plates in complete media. Cells were stimulated with IsdB for 24 h. After stimulation, cells were washed with PBS and harvested by gentle scraping. Cells were pelleted by centrifugation and fixed with a TEM-grade fixative buffer (4% paraformaldehyde in PBS) for 1 h at RT and subsequently stored at 4°C until processing.

For sample preparation, cells were washed three times for 5 min each with PBS, embedded in low-gelling agarose, and washed again three times for 5 min each with PBS. After dehydration in a graded ethanol series (30%, 50%, 70%, 90%, and 100% each for 30 min on ice), the material was infiltrated with the acryl resin LR White. For this, one part 100% ethanol was mixed with one part LR White and stored at 4°C overnight. After that, one part ethanol was mixed with two parts LR White for 2 h on ice followed by infiltration with pure resin for 6 h on ice, resin changing, and storage at 4°C overnight. The resin was left polymerized for 48 h at 60°C. Finally, samples were infiltrated with pure resin at RT. Ultrathin sections (50 nm) were cut on an ultramicrotome (Reichert Ultracut, Leica UK Ltd., Milton Keynes, UK) and picked up with Pioloform-coated hexagonal nickel grids before immunogold labeling.

For immunogold labeling, the flotation method was used. Briefly, the grids were placed with the sections face down on the droplets of washing or antibody solution at RT. The sections were incubated for 60 min on 5% (vol/vol) goat serum in incubation buffer [0.2% gelatine (wt/vol), 1% skim milk powder (wt/vol), and 0.1% Tween 20 in PBS], for 60 min on monoclonal anti-His_5_-antibody [diluted 1:50 in 0.5% bovine serum albumin (BSA) (wt/vol) in PBS, three times each 5 min on PBS, for 60 min on goat anti-mouse gold conjugates (5-nm diameter, Sigma-Aldrich) diluted 1:20 in 0.5% BSA (wt/vol) in PBS, and three times each 5 min on PBS]. Finally, sections were fixed with 1% glutaraldehyde in PBS for 5 min, washed on five droplets of deionized water each for 2 min, and stained with 4% aqueous uranyl acetate for 5 min. After blotting with filter paper, the grids were air dried and stored in a desiccator until examination under the microscope. The specimens were examined with a transmission electron microscope LEO 906 (Carl Zeiss Microscopy GmbH, Oberkochen, Germany) at an acceleration voltage of 80 kV. For image acquisition, a wide-angle dual-speed CCD camera Sharpeye (Tröndle, Moorenweis, Germany) was used, operated by ImageSP software. All micrographs were edited using Adobe Photoshop CS6.

### Scanning electron microscopy (SEM)

Human monocytes (1 × 10^6^/mL) were cultured on poly-L-lysine-coated coverslips in a 24-well plate in the presence or absence of IsdB for 24 h. After stimulation, the cells were washed with PBS and fixed in an SEM-grade fixative solution containing 2% glutaraldehyde and 2% paraformaldehyde in PBS for 1 h at RT and then stored at 4°C until further processing. The samples were washed three times for 5 min each with PBS and treated with 1% osmium tetroxide in PBS for 1 h. After washing three times for 5 min each with deionized water, the samples were subjected to dehydration in a graded series of aqueous ethanol solutions [10%, 30%, 50%, 70% (overnight), and 90%] for 10 min each step and the final three times for 10 min each in 100% ethanol before being critical point dried with liquid CO_2_ (K850, Quorum Technologies Ltd., UK). Finally, the specimens were mounted on aluminum stubs, sputtered with gold/palladium (SC 7640, Polaron Emitech), and examined with a scanning electron microscope EVO LS10 (Carl Zeiss Microscopy GmbH, Oberkochen, Germany). All micrographs were edited using Adobe Photoshop CS6.

### Microscale thermophoresis

Protein-protein interaction between rhTLR4 and IsdB or Lac-IsdB was addressed by MST. Thermophoresis is a biophysical technique that measures the affinity between two molecules in a solution by detecting variations in fluorescence signal as a result of a temperature change that is induced by an infra-red laser. The temperature influences the molecules’ interactions with the surrounding molecules. Higher temperature can lead to either attraction (positive thermophoresis) or repulsion (negative thermophoresis). All reagents, materials, and instruments were from NanoTemper technologies, Munich, Germany. Briefly, rhTLR4 was diluted in supplied labeling buffer to achieve a concentration of 20 µM (molar dye:protein ratio ≈ 3:1) and labeled using the protein labeling kit (RED-NHS 2nd generation) at RT for 30 min in the dark. The reactive NHS-ester groups in the dye bind covalently to primary amines (lysine residues) in the IsdB protein. Unbound dye was removed with the dye removal column equilibrated with MST buffer. The degree of labeling was determined using UV/VIS spectrophotometry at 650 nm and 280 nm. A degree of labeling of 0.8 was typically achieved. The labeled rhTLR4 protein was further diluted with assay buffer supplemented with 0.05% Tween 20 to 40 nM. Sixteen serial dilutions of IsdB or Lac-IsdB, concentrations ranging from 3.6 µM to 0.1 nM, were prepared using the same buffer. For the measurement, each IsdB dilution was mixed with an equal volume of labeled rhTLR4. The samples were loaded onto premium capillaries, and the MST was measured using a Monolith NT.115 instrument at an ambient temperature of 25°C. Instrument parameters were adjusted to 80% LED power and medium MST power. The MST data of both unbound and bound molecules were analyzed with MO Affinity Analysis software version 2.3 using the signal from an MST, on time of 15 s, and plotted as normalized fluorescence (Fnorm).

### Molecular docking of IsdB-TLR4

Briefly, docking was performed using the Hawkdock program (81), an integrated web server that combines docking poses, protein-protein docking, and identification of key residues between ligands. PDB files (IsdB: 5VMM and TLR4: 3FXI) were utilized through Hawkock. Sixteen potential complexes out of 100 initially modeled complexes were evaluated for complementary electrostatic surfaces, calculated as described before (82). Electrostatic potential was visually analyzed for the selected complexes between IsdB and TLR4 to assess the probability of complex formation. The interface between hTLR4 and IsdB of selected models was analyzed by DimPlot using LigPlot software to show hydrogen bonding and hydrophobic interaction between IsdB and TLR4 docked complexes (83).

### Production of human monoclonal antibodies against IsdB

Human monoclonal antibodies against IsdB were generated using a single B-cell cloning approach, following the method established by Wardemann and Kofer (84) with slight modifications. Briefly, PBMCs were isolated from the blood of healthy donors using density gradient centrifugation. B cells were purified and enriched using streptavidin nanobeads and a biotin-conjugated antibody mixture (biotin anti-CD2, CD3, CD14, CD15, CD16, CD36, CD56, CD123, and CD235ab). IsdB-specific B cells (CD3−/CD20+/CD14−/CD27+/IgD−/IsdB+) were sorted by fluorescence-activated cell sorting (Gallios Flow Cytometer, Beckman Coulter) using recombinant His-tagged IsdB. Total RNA was isolated from the single cells and reverse transcribed into cDNA. The antibody-coding gene sequences (variable region) of the heavy and light chains of the immunoglobulin (Ig) were amplified using PCR. Further amplification of the Ig-PCR products was conducted for subsequent sequencing of the variable regions of the Ig genes (Mix2Seq Kit; Eurofins Scientific SE). Meanwhile, the Ig-PCR products were cloned into expression vectors (AbVec2.0-IGHG1, IgG1; AbVec1.1-IGKC, Igκ; and AbVec1.1-IGLC2-XhoI, Igλ, Addgene). *E. coli* DC10B was transformed by the incorporation of the cloned plasmids.

Finally, human monoclonal antibodies were produced by transient transfection of Human Embryonic Kidney (HEK)-293T cells (ATCC, Manassas, USA), which were maintained in Dulbecco’s modified Eagle medium supplemented with 10% FCS and 1% PSG, with the plasmids of the matching heavy and light chains. The antibody was purified from the cell-free supernatant by affinity chromatography using a protein G-Sepharose column (Amersham). The concentration of the produced monoclonal antibody was determined by NanoDrop. It was subsequently characterized with respect to its antigen binding and putative neutralizing properties.

### IsdB-TLR4 binding assay

The binding affinity of IsdB to TLR4 was determined by ELISA. Briefly, 96-well microtiter plates (Nunc-Immuno plate, MaxiSorp, Thermo Fisher Scientific) were coated with 0.5 µg/mL recombinant TLR4 in coating buffer and left overnight at 4°. The plates were washed and subsequently blocked with 1% (wt/vol) BSA at RT for 1 h. The plates were then incubated for 2 h with either increasing concentrations of biotin-conjugated IsdB (1 to 20 µg/mL) or IsdB (1 µg/mL) alone or IsdB preincubated with increasing molar ratios of control mouse IgG or anti-IsdB antibodies at RT. The plates were washed and incubated with avidin IgG conjugated to HRP at RT for 1 h. After a final washing step, the signal was detected using the TMB substrate. Absorbance was measured at OD_450_ using a microplate reader (TECAN Infinite M200 plate reader).

### Statistics

All the data are presented as the mean ± SEM of indicated biological replicates (mBMDCs) or donors (human monocytes). Two-sample *t*-test was utilized to determine statistical significance between two groups. One-way ANOVA was utilized to determine statistical significance when more than two groups were involved. In some cases, two-way ANOVA was utilized to determine statistical significance between the stimulus or inhibitor + stimulus conditions. All statistical analyses were done using GraphPad Prism software version 9.0 (GraphPad, San Diego, California, USA).

## SUPPLEMENTAL MATERIAL

Supplemental material mbio.00225-23-s0001.docx Figures S1 to S6.

ASM does not own the copyrights to Supplemental Material that may be linked to, or accessed through, an article. The authors have granted ASM a non-exclusive, world-wide license to publish the Supplemental Material files. Please contact the corresponding author directly for reuse.
