# Supplementary material for: TLR4 sensing of IsdB of Staphylococcus aureus induces a proinflammatory cytokine response via the NLRP3-caspase-1 inflammasome cascade
Source: mBio. 2023 Dec 19;15(1):e00225-23. doi: 10.1128/mbio.00225-23 (PMC10790753; doi:10.1128/mbio.00225-23)
Supplement: Supplemental material — Figures S1 to S6. [file mbio.00225-23-s0001.docx]

# **Supplementary Information**

# **Manuscript title: TLR4 sensing of IsdB of *Staphylococcus aureus* induces a proinflammatory cytokine response via the NLRP3-Caspase-1 inflammasome cascade**

**Supplementary Figure 1**

**a b**

**Supplementary Fig. 1: IsdB induces IL-6 release in human monocytes in a dose- and time-dependent manner**. Primary human monocytes were stimulated with increasing concentrations of IsdB for 24 h (a) or with 10 µg of IsdB for the indicated time periods (b). Levels of IL-6 in the supernatants were quantified by ELISA. Data are the mean ± SEM of two independent experiments performed in technical triplicate. **p*<0.05, ***p*<0.01, ****p*<0.001 represents IsdB vs. untreated cells.

**Supplementary Figure 2**

**Supplementary Fig 2: A low concentration of LPS does not affect the IsdB-induced IL-6 release**. Human monocytes were isolated as described in the methods section. Different concentrations of LPS were applied alone or mixed with 10 µg/ml of IsdB, and the reaction mixture was incubated at 37 °C for 30 min. Cells were stimulated with increasing concentrations of LPS or LPS-spiked IsdB for 24 h. Levels of IL-6 were measured in the supernatants by ELISA. Data are the mean ± SEM of two independent experiments performed in duplicate, except for the 50 pg/mL concentration where the data were obtained based on monocytes from only one donor.

**Supplementary Figure 3**

**Supplementary Fig. 3: Denaturation of IsdB completely abrogated IL-6 release**. Monocytes were left unstimulated or stimulated with IsdB (10 µg/ml) or heat-inactivated-IsdB (95 °C for 30 min) for 24 h. Supernatants were analyzed for the indicated cytokines by multiplex assays. Data are presented as mean ± SEM of four independent experiments performed in technical triplicate. Each point represents one donor. One-way ANOVA was utilized to determine statistical significance. **p*<0.05, ***p*<0.01, *****p*<0.0001 represents IsdB vs. untreated cells. *^#^p*<0.05, *^##^p*<0.01, *^####^p*<0.0001 represents IsdB vs. heat-inactivated IsdB (IsdB-HI). ϕ represents unstimulated cells.

**Supplementary Figure 4**

**a**

**b**

**c**

**Supplementary Fig. 4: Effect of TLR4 blockade on IsdB-induced cytokine release**. Human monocytes were incubated in the presence or absence of anti-TLR4 antibody (5 µg/ml) (a) or CLI-095 (1 µM) (b) for 45 min before treatment with IsdB (10 µg/ml) (a and b) or LPS (100 ng/ml) (a) for an additional 24 h, n=4. Supernatants were analyzed for IL-6 by ELISA (a) or indicated cytokines by multiplex assay (b). (c) mBMDCs were incubated in the presence or absence of CLI-095 ((1 µM) for 45 min before treatment with Lac-IsdB (10 µg/ml) for an additional 6 h, n=3. IL-6 levels were determined by ELISA. Data are the mean ± SEM of four (a and b) and three (c) independent experiments performed in technical duplicate to triplicate. Each point represents one donor (a and b, human monocytes) or biological replicate (c, mBMDCs). Two-way ANOVA was utilized to determine statistical significance. **p*<0.05, ***p*<0.01, ***p<0.001, *****p*<0.0001 represents IsdB or Lac-IsdB vs untreated cells. *^#^p*<0.05, *^##^p*<0.01, *^###^p*<0.001, *^####^p*<0.0001 represents IsdB vs. IsdB+CLI-095 or TLR4 Ab. *^$$^p*<0.01 represents LPS vs. LPS+TLR4 Ab. CLI-095: TLR4 inhibitor, TLR4 Ab: anti-TLR4 IgG, n.s.: non-significant, ϕ represents respective controls or unstimulated cells.

**Supplementary Figure 5**

**Supplementary Figure 5: IsdB binding to TLR4.** An ELISA plate was coated overnight at 4°C with recombinant TLR4 (0.5 µg/ml) and incubated with increasing concentrations of biotin-conjugated recombinant IsdB (rIsdB), ranging from 1 to 20 µg/ml. The binding of IsdB to TLR4 was detected with Avidin-IgG conjugated to HRP and the TMB substrate. The absorbance (OD450) was measured using a microplate reader. The data represents the mean ± SEM of three independent experiments performed in triplicate.

**Supplementary Figure 6**

**
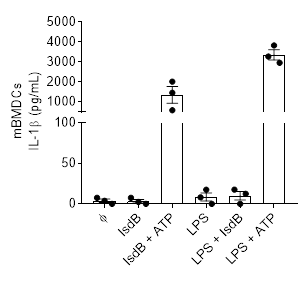
**

**Supplementary Fig. 6: IsdB induces priming but does not contribute to activation of the inflammasome.** Murine bone marrow-derived dendritic cells (mBMDCs) were left unprimed or primed with either IsdB (10 µg/ml) or LPS (100 ng/ml) for 3 h followed by treatment with ATP (5 mM) or IsdB (10 µg/ml) for additional 6 h. Cell-free supernatants were analyzed for IL-1β release by ELISA. Data are the mean ± SEM of three independent experiments performed in duplicate to triplicate. ATP: Adenosine triphosphate, n.s.: non-significant. ϕ represents respective controls or unstimulated cells.
